# Effect of perioperative glucocorticoid administration on postoperative complications following esophagectomy: A meta-analysis

**DOI:** 10.3892/ol.2013.1748

**Published:** 2013-12-10

**Authors:** QIANG GAO, HSIAO-PEI MOK, WEN-PING WANG, XIAO-FEIZUO, LONG-QI CHEN

**Affiliations:** 1 West China Medical School, Sichuan University, Chengdu, Sichuan 610041, P.R. China; 2 Department of Thoracic Surgery, West China Hospital, Sichuan University, Chengdu, Sichuan 610041, P.R. China

**Keywords:** esophageal neoplasms, postoperative complications, glucocorticoids

## Abstract

Perioperative corticosteroid administration is a controversial therapy for improving the short-term prognosis following surgery. The objective of the current meta-analysis was to evaluate the effects of the perioperative use of corticosteroids during esophagectomy for esophageal carcinoma. A comprehensive study was performed using references selected from the Cochrane Central Register of Controlled Trials (CENTRAL), PubMed, MEDLINE (Ovid databases), EMBASE and three Chinese databases (Chinese Biomedical Literature Database, Chinese National Knowledge Infrastructure and VIP Database for Chinese Technical Periodicals). Eligible studies were restricted to randomized clinical trials that reported data from patients undergoing esophagectomy. In addition, treated groups of patients received perioperative corticosteroid administration and control groups received a placebo infusion, such as saline water. The studies evaluated the incidence of postoperative complications and the variation of inflammatory mediators. All extracted data underwent meta-analysis using Review Manager 5.1 software. Only six studies were eligible for selection. The following parameters were found to be reduced following the use of methylprednisolone: Interleukin (IL)-6 immediately following surgery and on postoperative days (PODs) 1 and 3; IL-8 immediately following surgery; and PaO_2_/FiO_2_ on POD 3. Moreover, organ failure, cardiovascular complications and pulmonary morbidity were all reduced in patients with corticosteroid usage. Certain factors showed no significant differences between the treated and control groups, including IL-8 on POD 1, IL-6 prior to surgery and on POD 5, PaO_2_/FiO_2_ following surgery, mortality, anastomotic leakage, severe infection and renal and hepatic failure. Prophylactic administration of methylprednisolone during the perioperative period may reduce the incidence of specific types of postoperative complications and inhibit the postoperative inflammatory reaction. Additional randomized controlled trials must be performed.

## Introduction

As an invasive procedure, surgery for esophageal carcinoma may lead to serious inflammatory reactions and is characterized by extreme changes in the serum level of cytokines and acute phase proteins, particularly interleukin (IL)-6 and polymorphonuclear neutrophil elastase (1). It is generally well accepted that excessive inflammation is detrimental to postoperative recovery, therefore, the use of perioperative corticosteroid therapy to inhibit inflammatory mediators has been recommended as an approach to improve prognosis (2,3). However, as reported by Yano *et al*(4), the clinical benefits and risks associated with the administration of preoperative steroid therapy remain unclear due to controversial study results and a lack of thorough investigation. A number of surgeons also express concern with regard to delayed wound healing and the potential for tumor recurrence following corticosteroid administration in routine clinical work. Consequently, perioperative corticosteroid administration has not been widely accepted or used. The present meta-analysis study aims to investigate the efficacy and safety of perioperative corticosteroid administration following esophagectomy.

## Materials and methods

### Inclusion criteria and outcomes

To avoid potential bias, the present meta-analysis only included randomized clinical trials. Participants must have undergone an esophagectomy for a pathologically-confirmed esophageal carcinoma. Trials must have included an intervention group, which received perioperative corticosteroid administration, and a control group, which received a placebo infusion of an inert substance, such as saline water. Data from eligible studies were extracted by two independent authors following a protocol accepted by all authors. Extracted information included population data, number of participants in each group, number of patients that preliminarily withdrew or dropped out, completeness of follow-up data, corticosteroid administration route, dosage and patient outcomes. The primary outcomes were mortality and morbidity, including pulmonary disorders, severe infection, anastomotic leakage, renal and liver failure, cardiovascular disorders, failure of any organ and additional adverse reactions, such as altered postoperative plasma levels of IL-6 or −8 and lower postoperative PaO_2_/FiO_2_ ratios.

### Literature search sources

A comprehensive search was performed to identify all relevant studies from the electronic and printed literature. All included studies were analyzed regardless of the language used. The key words used for identifying the studies included prednisone, prednisolone, methylprednisolone, glucocorticoid, hydrocortisone, corticosteroid, esophagectomy, esophageal cancer and randomized controlled trial. The following bibliographic databases were searched: PubMed (up to February 6, 2013), the Cochrane Central Register of Controlled Trials (CENTRAL; up to February 6, 2013), MEDLINE (between 1946 and January 31, 2013), EMBASE (between 1974 and February 6, 2013), the Chinese Biomedical Literature Database (up to February 6, 2013), Chinese National Knowledge Infrastructure (up to February 6, 2013) and the VIP Database for Chinese Technical Periodicals (up to February 6, 2013).

### Statistical analysis

Two authors selected the relevant studies by searching publication titles and abstracts. All the extracted data underwent meta-analysis using Review Manager 5.1 software (The Cochrane Collaboration, Copenhagen, Denmark). The Mantel-Haenszel method was used to analyze dichotomous data and the risk ratio (RR) had 95% confidence intervals (CI). For continuous data, the inverse variance method was used and mean differences with 95% CIs were expressed.

The clinical and methodological heterogeneity were initially assessed. The χ^2^ test was used to analyze statistical heterogeneity, and statistical significance was indicated by a value of P<0.1. The I^2^ test was also used to estimate the total variation across all included studies. The level of heterogeneity, which determined whether a random-effects model or a fixed-effects model was used for pooled data analysis, was judged according to the recommendations of Higgins and Green (5). The risk of bias was assessed according to criteria described in the Cochrane Handbook for Systematic Reviews of Interventions (5). The level of evidence quality was assessed using the Grading of Recommendations Assessment, Development and Evaluation (GRADE) profiler software (version 3.2 for Windows; developed by Jan Brozek, Andrew Oxman and Holger Schünemann, 2008).

## Results

After analyzing all studies retrieved using the key word search, only six eligible studies were selected, all of which were Japanese and used methylprednisolone. In addition, no treatments were administered orally or following the completion of surgery. The characteristics of the included studies are listed in Table I.

**Figure 1:**
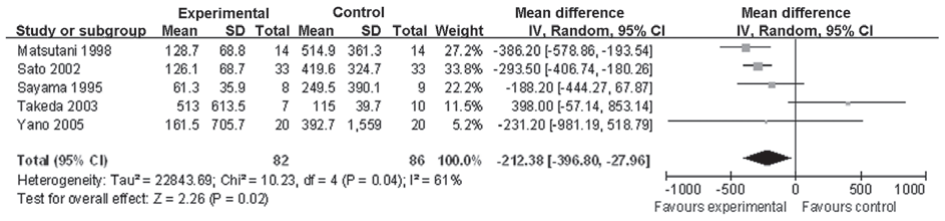
Postoperative levels of IL-6. A weighted mean difference of <0 indicated a lower plasma concentration in the methylprednisolone group compared with the control group. IL-6, interleukin-6; SD, standard deviation; CI, confidence interval; df, degrees of freedom.

**Figure 2:**
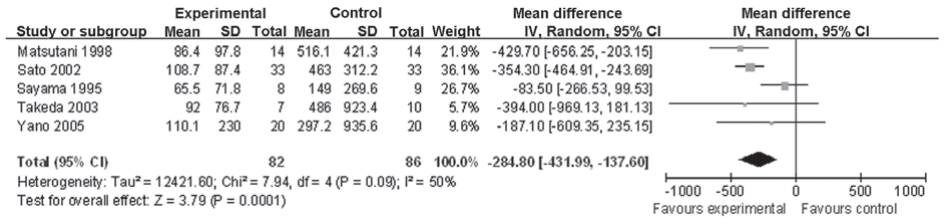
Levels of IL-6 on POD 1. A weighted mean difference of <0 indicated a lower plasma concentration in the methylprednisolone group compared with the control group. SD, standard deviation; CI, confidence interval; df, degrees of freedom; POD, postoperative day.

**Figure 3:**
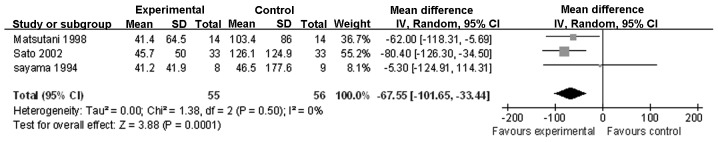
Levels of IL-6 on POD 3. A weighted mean difference of <0 indicated a lower plasma concentration in the methylprednisolone group compared with the control group. SD, standard deviation; CI, confidence interval; df, degrees of freedom; POD, postoperative day.

**Figure 4:**
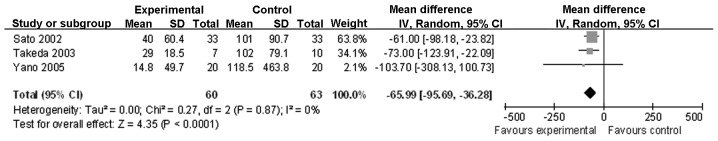
Postoperative levels of IL-8. A weighted mean difference of <0 indicated a lower plasma concentration in the methylprednisolone group compared with the control group. SD, standard deviation; CI, confidence interval; df, degrees of freedom.

**Figure 5:**
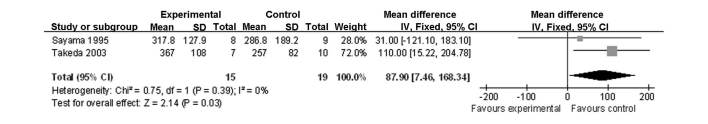
Levels of PaO_2_/FiO_2_ on POD 3. A weighted mean difference of <0 indicated a larger PaO_2_/FiO_2_ ratio in the methylprednisolone group compared with the control group. SD, standard deviation; CI, confidence interval; df, degrees of freedom; POD, postoperative day.

**Figure 6:**
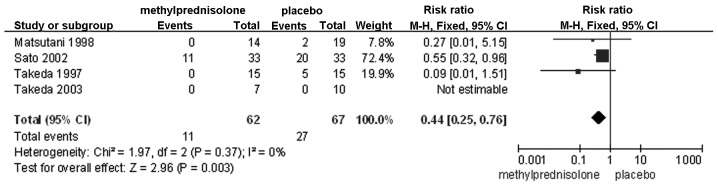
Postoperational incidence of organ failure. A risk ratio of <1 indicated fewer adverse reactions in the methylprednisolone group compared with the control group. CI, confidence interval; df, degrees of freedom.

**Figure 7:**
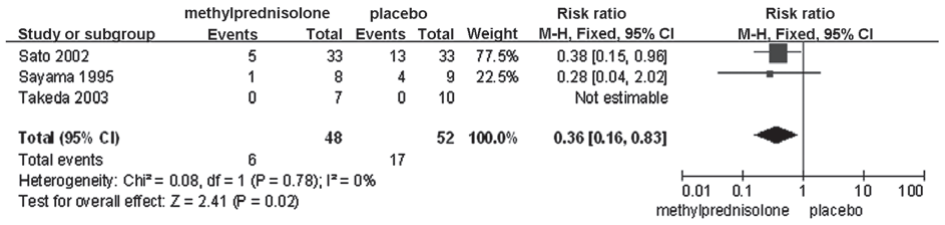
Postoperational incidence of cardiovascular disorder. A risk ratio of <1 indicated fewer adverse reactions in the methylprednisolone group compared with the control group. CI, confidence interval; df, degrees of freedom.

**Figure 8:**
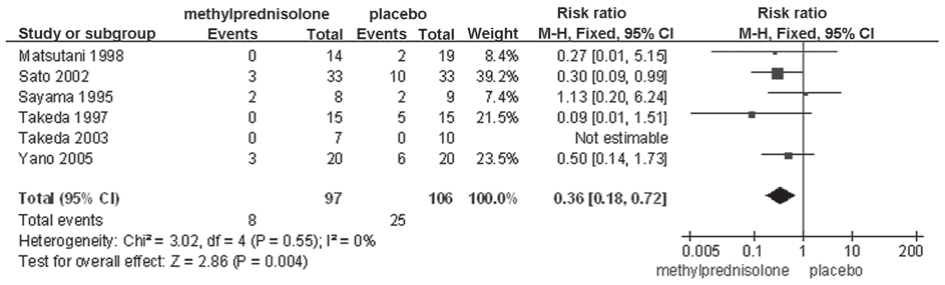
Postoperational incidence of pulmonary disorder. A risk ratio of <1 indicated fewer adverse reactions in the methylprednisolone group compared with the control group. CI, confidence interval; df, degrees of freedom.

The following parameters showed significant differences between the control and methylprednisolone-treated groups, as demonstrated in the following figures: IL-6 following surgery (Fig. 1); IL-6 on postoperative day (POD) 1 (Fig. 2); IL-6 on POD 3 (Fig. 3); IL-8 following surgery (Fig. 4); PaO_2_/FiO_2_ on POD 3 (Fig. 5); failure of any organ (Fig. 6); cardiovascular disorders (Fig. 7); and pulmonary disorders (Fig. 8). The remaining factors showed no significant differences, notably, IL-8 on POD 1, IL-6 prior to surgery, IL-6 on POD 5, PaO_2_/FiO_2_ following surgery, mortality, anastomotic leakage, severe infection and renal and hepatic failure (Table II).

Following evaluation of the GRADE profile, the quality of evidence was acceptable for the description of postoperative complications, including anastomotic leakage, organ failure, severe infection and pulmonary disorders. By contrast, for mortality, cardiovascular disorders, renal and hepatic failure, inflammatory cytokines and the PaO_2_/FiO_2_ ratio, the evidence was significantly weaker (Table III).

## Discussion

As one of the more radical therapies for esophageal cancer, esophagectomy is associated with a high incidence of postoperative complications (6). In addition, esophagectomy is stressful and induces an aggressive inflammatory response (7). There appears to be a plausible correlation between high levels of inflammation and the incidence of postoperative complications (8). It is well known that postoperative immunological function, particularly cell-mediated immunity, is profoundly repressed by an excessive inflammatory response (9). Nekhaev *et al*(10) reported that prophylactic administration of granulocytic colony-stimulating factor reduced the incidence of specific postoperative complications, as well as the length of hospitalization. In this respect, maintaining a sufficient inflammatory stress reaction may modulate the patient's levels of immunity in a way that it is beneficial for recovery. Consistent with this concept, Sato *et al*(11) and Shimada *et al*(2) reported that the perioperative administration of methylprednisolone restricted inflammatory cytokines to a moderate level and improved the postoperative clinical course. The present study was designed to highlight a comprehensive meta-analysis of the efficacy and safety of perioperative corticosteroid administration, associated with recovery from esophagectomy.

A predominant observation of the current study was that corticosteroid treatment decreased the levels of postoperative inflammatory molecules. For example, while the preoperative levels of IL-6 were not different between the control and methylprednisolone-treated groups, the postoperative IL-6 levels in patients treated with methylprednisolone were significantly lower on PODs 1 and 3. A similar change was observed for the postoperative levels of IL-8. This is likely to be attributed to the evidence that glucocorticoids are potent anti-inflammatory agents that inhibit the activity of a number of immunoregulatory genes (12), including nuclear κB (12,13). An additional mechanism hypothesized by Munck *et al*(14) states that glucocorticoids stabilize the lysosome membrane and contain these molecules.

Takeda *et al*(15) reported a negative correlation between the levels of IL-8 and the PaO_2_/FiO_2_ ratio in bronchoalveolar lavage fluid. A previous study also reported that IL-8 may be important for increasing the permeability of the pulmonary endothelium through the activation of neutrophils that generate toxic agents, including hyperoxide and protease (16). Therefore, in theory, once IL-8 levels are suppressed by methylprednisolone, pulmonary function must improve. The results of the current meta-analysis showed significant differences in the PaO_2_/FiO_2_ ratio between the control and methylprednisolone-treated groups on POD 3, with a higher ratio in the treated group. By contrast, the postoperative PaO_2_/FiO_2_ ratio was not significantly different between the groups on POD 1. The decrease in the oxygenation index following surgery is likely to be associated with a systemic inflammatory response, lung injury and/or ischemic reperfusion injury of the pulmonary vasculature. This, in turn, may activate neutrophils to generate toxic substances and result in further lung injury, thickening of the respiratory membrane and increased pulmonary endothelium permeability. Although a preoperative single dose of methylprednisolone does not completely buffer the stress resulting from all these injurious factors, the repression of inflammatory cytokines by the steroid is clear. This effect may ultimately lead to a decrease in the incidence of postoperative pulmonary disorders, a hypothesis that is consistent with the present meta-analysis.

In addition, it must be noted that the IL-6 levels on POD 5 and the IL-8 levels on POD 1 showed no significant differences between the control and methylprednisolone-treated groups. This was likely to be due to the administration of only a single dose of methylprednisolone in all the trials and as methylprednisolone exhibits a relatively short half-life of ~2.8 h in blood. Thus, with decreasing drug concentration, the anti-inflammatory effect is likely to decrease within a few hours to days following surgery. Yeager *et al*(17) also proposed that the dose-dependent effects of anti-inflammatory agents are likely to be more prominent. These conclusions indicate that preoperative administration of methylprednisolone alone is not sufficient to attain the highest degree of anti-inflammatory effects and that perioperative administration must be considered.

Of the included studies in the present meta-analysis, the patients with postoperative cardiovascular disorders all exhibited underlying conditions, including abnormal changes in the preoperative electrocardiogram, and an elderly age (18). Surgical manipulation directly irritates the heart, particularly in a procedure such as an esophagectomy (19). In addition, postoperative hypoxemia caused by conditions, including low oxygenation index or pulmonary complications, is a crucial factor in the pathogenesis of cardiovascular disorders (20). As discussed, the administration of methylprednisolone has been hypothesized to alleviate excessive systemic inflammation and improve the oxygenation index and pulmonary function. Thus, it is likely that is also decreases the rate of postoperative cardiovascular disorders. The results of the present meta-analysis showed a significant difference in the incidence of these disorders between the control and methylprednisolone-treated groups, with a lower incidence in the treated group.

Postoperative organ failure is attributable to multiple etiological factors, notably severe infection, serious trauma and sepsis, which are all factors that activate the inflammatory cascade (21). If this response is not repressed, organ failure is likely to occur in any organ, thus illustrating the usefulness of perioperative administration of an anti-inflammatory agent, such as methylprednisolone. The current meta-analysis showed that morbidity associated with organ failure was lower in the methylprednisolone-treated group compared with the control group, with the exception of renal and hepatic failure, which showed no significant difference. This clear discrepancy is hypothesized to be due to the data associated with organ failure, as it was assessed in the present meta-analysis by combining data from all organs, which is likely to magnify the effect. Furthermore, the anastomotic leakage and mortality rates were similar in the groups, indicating that the use of methylprednisolone is likely to be well tolerated. However, since a few of the trials that were included had small quantities of participants, specific clinical differences may not have been detected.

As aforementioned, there are multiple predisposing causes of postoperative complications, among which hypernomic inflammation is significant. Nevertheless, a moderate inflammatory response is indispensable for postoperative recovery, particularly when severe infection occurs (22). Thus, it is crucial to maintain a delicate balance between pro- and anti-inflammation. The present meta-analysis showed that the rate of severe infection between the control and methylprednisolone-treated groups was similar. The dose of methylprednisolone that was used in the included trials varied between 250 mg/body and 30 mg/kg. Calandra *et al* reported that low concentrations of glucocorticoids may activate the secretion of macrophage migration inhibitory factor (MIF) by macrophages (23), and the proinflammatory effects of MIF are then able to overcome the anti-inflammatory effects of the steroids (24). Furthermore, Gao *et al* and Donnelly *et al* found that a high concentration of MIF in alveoli contributes to acute respiratory distress syndrome (25,26). From these previous studies, it appears that certain physiological mechanisms of methylprednisolone remain to be elucidated (27), with a clear requirement for future investigation of the administration time and optimal dosage.

In conclusion, the present meta-analysis indicates that methylprednisolone treatment may be associated with reduced levels of the IL-6 and −8 inflammatory cytokines and higher PaO_2_/FiO_2_ ratios by POD 3. However, this association requires confirmation due to the smaller size and a lack of rigorous randomized controlled design in a number of the included studies. A marked association was demonstrated in the administration of methylprednisolone with a lower incidence of organ failure and pulmonary disorder. One significant cause of heterogeneity is the variation in dosage and time of administration, which weakened the evidence quality. Thus, future rigorous randomized controlled trials with a greater number of participants are likely to be useful for clarifying the conclusions of the current meta-analysis and for determining the optimal administration time and dosage of methylprednisolone.

## Figures and Tables

**Table I:** Characteristics of the studies included in the present meta-analysis.

First author (year) [ref]	Placebo (dose)	Intervention (dose)	Administration time	Intervention group, n	Placebo group, n
Sato *et al*(2002) [11]	Saline (10 mg/kg)	Methylprednisolone (10 mg/kg)	Within 30 min prior to surgery	33	33
Yano *et al*(2005) [4]	Saline (500 mg/body)	Methylprednisolone (500 mg/body)	Within 2 h prior to surgery	20	20
Matsutani *et al*(1998) [25]	Saline (10 mg/kg)	Methylprednisolone (10 mg/kg)	At the time of induction of anesthesia	14	19
Takeda *et al*(2003) [15]	Saline (10 mg/kg)	Methylprednisolone (10 mg/kg)	Prior to induction of anesthesia	7	10
Takeda *et al*(1997) [26]	Saline (30 mg/kg)	Methylprednisolone (30 mg/kg)	Prior to induction of anesthesia	15	15
Sayama *et al*(1995) [27]	Saline (250 mg/body)	Methylprednisolone (250 mg/body)	Within 2–3 h prior to surgery	8	9

**Table II:** Non-significant outcomes associated with methylprednisolone treatment.

Outcome	Studies, n	Participants, n	Effect estimate, mean (range)	P-value
IL-8 on POD 1^a^	3	123	−15.73 (−34.64–3.18)	0.10
IL-6 prior to surgery^a^	3	111	1.73 (−16.22–19.68)	0.85
IL-6 on POD 5^a^	2	94	−54.95 (−140.70–30.79)	0.21
PaO_2_/FiO_2_ following surgery^a^	2	34	−3.77 (−85.94–78.40)	0.93
PaO_2_/FiO_2_ on POD 1^a^	2	34	−44.88 (−115.77–26.01)	0.21
Mortality^b^	2	96	0.13 (0.01–2.12)	0.15
Anastomotic leakage^c^	5	186	0.73 (0.26–2.07)	0.56
Severe infection^c^	5	186	0.57 (0.23–1.38)	0.21
Renal failure^c^	3	113	0.79 (0.34–1.85)	0.59
Hepatic failure^c^	3	113	0.38 (0.09–1.56)	0.18

Effects were determined as the ^a^mean difference (95% CI), ^b^Peto odds ratio (95% CI) and ^c^risk ratio (95% CI). POD, postoperative day; CI, confidence interval; IL, interleukin.

**Table III:** Quality of evidence assessed by GRADE profile.

								Summary of observations					
			
	Quality assessment							Patients, n (%)		Effect			
					
Outcome	Studies, n	Design	Limitations	Inconsistency	Indirections	Imprecision	Other considerations	Study	Control	RR (95% CI)	Absolute (range)	Quality	Importance
IL-6 prior to surgery	3	Randomized trial	Serious^a^,^b^	Not serious	Not serious	Serious^c^	Reporting bias^d^	55	56	-	MD 1.73 (−16.22 to 19.68)	Extremely low	Important
IL-6 following surgery	5	Randomized trial	Serious^a^,^b^	Serious^e^	Not serious	Not serious	None	82	86	-	MD 212.38 (−396.8 to 27.96)	Low	Important
IL-6 on POD 1	5	Randomized trial	Serious^a^,^b^	Serious^f^	Not serious	Not serious	None	82	86	-	MD −284.8 (−431.99 to −137.61)	Low	Important
IL-6 on POD 3	3	Randomized trial	Serious^a^,^b^	Not serious	Not serious	Not serious	Reporting bias^g^	55	56	-	MD −67.55 (−101.65 to −33.44)	Low	Important
IL-6 on POD 5	2	Randomized trial	Serious^a^,^b^	Serious^h^	Not serious	Serious^c^,^i^	Reporting bias^j^	47	47	-	MD −54.95 (−140.7 to 30.79)	Extremely low	Important
IL-8 following surgery	3	Randomized trial	Serious^a^,^b^	Not serious	Not serious	Not serious	Reporting bias^d^	60	63	-	MD −65.99 (−95.69 to −36.28)	Low	Important
IL-8 on POD 1	3	Randomized trial	Serious^a^,^b^	Not serious	Not serious	Serious^c^	Reporting bias^d^	60	63	-	MD −15.73 (−34.64 to 3.18)	Extremely low	Important
PaO_2_/FiO_2_ following surgery	2	Randomized trial	Serious^a^,^k^	Not serious	Not serious	Serious^c^,^i^	Reporting bias^j^	15	19	-	MD −3.77 (−85.94 to 78.4)	Extremely low	Critical
PaO_2_/FiO_2_ on POD 1	2	Randomized trial	Serious^a^,^k^	Not serious	Not serious	Serious^c^,^i^	Reporting bias^j^	15	19	-	MD −44.88 (−115.77 to 26.01)	Extremely low	Critical
PaO_2_/FiO_2_ on POD 3	2	Randomized trial	Serious^a^,^b^	Not serious	Not serious	Serious^i^	Reporting bias^j^	15	19	-	MD 87.9 (7.46 to 168.34)	Extremely low	Critical
Anastomotic leakage	5	Randomized trial	Serious^a^,^b^	Not serious	Not serious	Not serious^c^	None	5/89 (5.6)	7/97 (6.1)	0.73 (0.26–2.07)	16 fewer/1,000	Moderate	Critical
Mortality	2	Randomized trial	Serious^a^,^b^	Not serious	Not serious	Serious^c^	Reporting bias^j^	0/48 (0)	2/48 (6.7)	0.13 (0.01–2.12)	57 fewer/1,000	Extremely low	Critical
Any organ failure	4	Randomized trial	Serious^a^,^b^	Not serious	Not serious	Serious^l^	Strong association^m^	11/62 (17.7)	27/67 (21.9)	0.44 (0.25–0.76)	122 fewer/1,000	Moderate	Critical
Severe infection	5	Randomized trial	Serious^a^,^b^	Not serious	Not serious	Not serious	None	6/89 (6.7)	11/97 (6.1)	0.57 (0.23–1.38)	26 fewer/1,000	Moderate	Critical
Pulmonary disorder	6	Randomized trial	Serious^a^,^b^	Not serious	Not serious	Not serious	None	8/97 (8.2)	25/106 (26.1)	0.36 (0.18–0.72)	167 fewer/1,000	Moderate	Critical
Cardiovascular failure	3	Randomized trial	Serious^a^,^b^	Not serious	Not serious	Serious^d^	Reporting bias^n^	6/48 (12.5)	17/52 (39.4)	0.36 (0.16–0.83)	252 fewer/1,000	Extremely low	Critical
Renal failure	3^a^,^b^	Randomized trial	Serious^a^,^b^	Not serious	Not serious	Serious^c^,^d^	Reporting bias^n^	7/55 (12.7)	9/58 (6.7)	0.79 (0.34–1.85)	14 fewer/1,000	Extremely low	Critical
Hepatic failure	3	Randomized trial	Serious^a^,^b^	Not serious	Not serious	Serious^c^,^d^	Reporting bias^n^	2/55 (3.6)	6/58 (6.7)	0.38 (0.09–1.56)	41 fewer/1,000	Extremely low	Critical

a Awareness of allocation concealment is unknown in all included studies;

b only one trial was performed as a single blind procedure and all trials did not have a blind outcome assessor;

c the CI was too wide due to the small quantity of participants;

d only three small studies were included;

e I^2^=61%, there was moderate heterogeneity;

f I^2^=50%, there may be moderate heterogentiy;

g only three small trials were included;

h I^2^=58%, there may be moderate heterogeneity;

i only two small trials were included;

j only two trials included;

k two trials were performed and not by blind procedure and all trials did not have a blind outcome assessor;

l small quantity of participants in the included studies;

m RR=0.44 and 95% CI, 0.25–0.76;

n only three studies were included.

GRADE, Grading of Recommendations Assessment, Development and Evaluation; RR, risk ratio; MD, mean difference; CI, confidence interval; IL, interleukin.
